# Spin Ordering Induced Broadband Photodetection Based on Two‐Dimensional Magnetic Semiconductor *α*‐MnSe

**DOI:** 10.1002/advs.202202177

**Published:** 2022-06-05

**Authors:** Nan Zhou, Zhimiao Zhang, Fakun Wang, Junhao Li, Xiang Xu, Haoran Li, Su Ding, Jinmei Liu, Xiaobo Li, Yong Xie, Rusen Yang, Ying Ma, Tianyou Zhai

**Affiliations:** ^1^ School of Advanced Materials and Nanotechnology Xidian University Xi'an 710126 P. R. China; ^2^ Guangzhou Institute of Technology Xidian University Guangzhou 710068 P. R. China; ^3^ State Key Laboratory of Materials Processing and Die and Mould Technology School of Materials Science and Engineering Huazhong University of Science and Technology Wuhan 430074 P. R. China; ^4^ School of Electrical and Electronic Engineering Nanyang Technological University Singapore 639798 Singapore; ^5^ Institute of Information Sensing Xidian University Xi'an 710126 P. R. China

**Keywords:** *α*‐MnSe, broadband photodetection, magnons, spin ordering, two‐dimensional magnetic semiconductors

## Abstract

Two‐dimensional (2D) magnetic semiconductors are considered to have great application prospects in spintronic logic devices, memory devices, and photodetectors, due to their unique structures and outstanding physical properties in 2D confinement. Understanding the influence of magnetism on optical/optoelectronic properties of 2D magnetic semiconductors is a significant issue for constructing multifunctional electronic devices and implementing sophisticated functions. Herein, the influence of spin ordering and magnons on the optical/optoelectronic properties of 2D magnetic semiconductor *α*‐MnSe synthesized by space‐confined chemical vapor deposition (CVD) is explored systematically. The spin‐ordering‐induced magnetic phase transition triggers temperature‐dependent photoluminescence spectra to produce a huge transition at Néel temperature (*T*_*N*_ ≈ 160 K). The magnons‐ and defects‐induced emissions are the primary luminescence path below *T*_*N*_ and direct internal ^4^_a_T_1g_→^6^A_1g_ transition‐induced emissions are the main luminescence path above *T*_*N*_. Additionally, the magnons and defect structures endow 2D *α*‐MnSe with a broadband luminescence from 550 to 880 nm, and an ultraviolet–near‐infrared photoresponse from 365 to 808 nm. Moreover, the device also demonstrates improved photodetection performance at 80 K, possibly influenced by spin ordering and trap states associated with defects. These above findings indicate that 2D magnetic semiconductor *α*‐MnSe provides an excellent platform for magneto‐optical and magneto‐optoelectronic research.

## Graphical abstract

2D magnetic semiconductor *α*‐MnSe flakes are synthesized by space‐confined CVD. Impressively, the spin‐ordering‐related magnons and defects confirmed by low‐temperature photoluminescence spectra confer themselves with a broadband luminescence from 550 to 880 nm, an ultraviolet–near‐infrared photoresponse from 365 to 808 nm, and enhanced photon‐to‐electron conversion performance at 80 K.

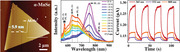

## 1 Introduction

Two‐dimensional (2D) magnetic semiconductors are considered to have great application prospects in spintronic logic devices, memory devices, and photodetectors, due to their special structures and exceptional physical properties in 2D confinement including the ability to control spin orientation and charge dynamics in elementary particles.^[^ ^1^ ^]^ For instance, 2D Cr_2_Ge_2_Te_6_ exhibited intrinsic ferromagnetism in atomic layers;^[^ ^2^ ^]^ 2D Cr_2_S_3_ processed ferromagnetism with a maximum saturation magnetic momentum of 65 μemu.^[^ ^3^ ^]^ For 2D magnetic semiconductors, understanding the influence of magnetism (such as spin ordering and magnons) on their optical/optoelectronic properties is a significant issue for constructing multifunctional electronic devices and implementing sophisticated functions.

Recently, a series of studies involving magneto‐optical and magneto‐electrical were reported, such as helical luminescence in monolayer CrI_3_ with helicity determined by underlying magnetic order;^[^ ^4^ ^]^ magnetic‐controlled anisotropic Raman scattering;^[^ ^5^ ^]^ tunneling magnetoresistance related to layer‐by‐layer antiferromagnetic ordering,^[^ ^6^ ^]^ metamagnetic transition,^[^ ^7^ ^]^ and multiple transitions to different magnetic states;^[^ ^8^ ^]^ controlling magnetism by electrostatic doping;^[^ ^9^ ^]^ and light helicity detector with helicity‐selective photoresponse determined by magnetic state;^[^ ^10^ ^]^ confirming that magnetic property and optical/electrical properties of 2D CrI_3_ interact and influence each other. These research progress suggested 2D magnetic semiconductors provide an excellent platform for exploring light–matter interactions and studying magneto‐optical and magneto‐electrical phenomena in 2D limit, and further emphasized that understanding the coupling mechanism between optoelectronic properties and magnetic property is of great significance to develop spin‐optoelectronic devices and multifunctional optoelectronic devices. However, up to now, the influence of spin ordering and magnons on optical/optoelectronic properties is still rarely reported, which obviously hinders further studying the physical properties of 2D magnetic semiconductors and revolutionizing current optical and optoelectronic applications. Therefore, continuing to expand the library of 2D magnetic semiconductors and systematically exploring the influence of spin ordering and magnons on their optical/optoelectronic properties are necessary for discovering new physical phenomena and enabling sophisticated functionality.

*α*‐MnSe is a magnetic semiconductor with a wide bandgap of ≈3.2 eV,^[^ ^11^ ^]^ which possesses a series of interesting properties including unique antiferromagnetic ordering with a large magnetic moment,^[^ ^12^ ^]^ nanoparticle‐size‐dependent bandgaps that cover ultraviolet to visible range.^[^ ^13^ ^]^ Therefore, it can be applied to many aspects such as diluted magnetic semiconductors, field‐effect transistor (FET), magnetic‐optical devices, and optoelectronic devices.^[^ ^14^ ^]^ Moreover, owing to the presence of spin‐ordering‐induced magnetic phase transition at the Néel temperature (130 K < *T*_*N*_ < 197 K) and intrinsic magnons in the crystalline structures,^[^ ^15^ ^]^ *α*‐MnSe is supposed to yield novel optical properties and abnormal device performance.^[^ ^11^, ^16^ ^]^ However, due to the intrinsic isotropic chemical bondings in 3D directions,^[^ ^17^ ^]^ enormous difficulties and challenges will be existed in the preparation of 2D *α*‐MnSe flakes. Until now, 2D *α*‐MnSe has only been realized by very few research groups,^[^ ^18^ ^]^ and the effects of spin ordering and magnons on its optical and photoelectric properties have not yet been explored, which is not conducive to exploring its novel properties and unique potential applications.

In this work, 2D magnetic semiconductor *α*‐MnSe flakes were obtained via space‐confined chemical vapor deposition (CVD). On this basis, the influence of spin ordering and magnons on their optical/optoelectronic properties was further systematically explored. The luminescence performance was investigated via temperature‐dependent photoluminescence (PL) spectra, which cover a broadband spectrum ranging from 550 to 880 nm, attributed to magnons‐ and defects‐induced super‐bandgap emission. The spin‐ordering‐induced magnetic phase transition triggers PL spectra to produce a huge transition point—magnons‐ and defects‐induced emissions were the main luminescence pathway below *T*_*N*_ (≈160 K), and direct internal ^4^_a_T_1g_→^6^A_1g_ transition‐induced emissions were the primary luminescence pathway above *T*_*N*_. Stemming from the existence of inherent magnons and defects, 2D *α*‐MnSe flakes exhibit broadband photoresponse from 365 to 808 nm with a high responsivity (*R*_*λ*_) of 521.8 A W^−1^ and an excellent detectivity (D*) of 3.46 × 10^11^ Jones. In addition, the device demonstrates a superior photocurrent‐optical power fitting factor and response time at 80 K to those at 300 K, possibly influenced by spin ordering and trap states associated with defects. In short, the precise synthesis of 2D *α*‐MnSe flakes has further flourished the library of 2D magnetic semiconductors, and the influence mechanism of spin ordering and magnons on its optical/optoelectronic properties were basically elucidated, enriching the magneto‐optical and magneto‐electrical research. These above findings indicated that 2D magnetic semiconductor *α*‐MnSe has great application potential in magneto‐optical and magneto‐optoelectronic devices.

## 2 Results and Discussion

**Figure 1:**
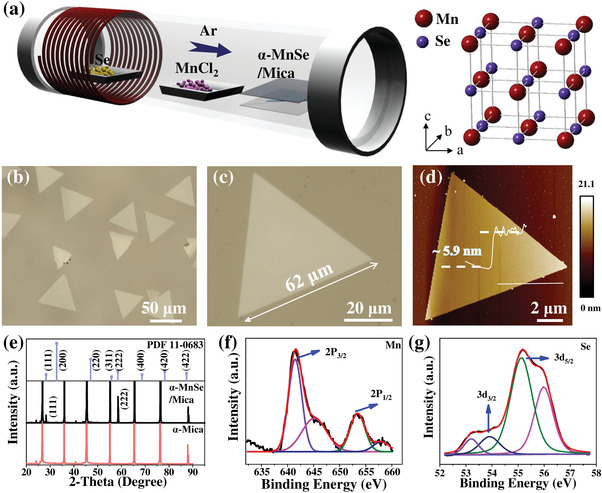
Preparation and characterizations of 2D *α*‐MnSe flakes grown by CVD. a) Preparation schematic and the structural model of nonlayered *α*‐MnSe. b) Optical image of prepared triangle‐shaped *α*‐MnSe samples. c) A typical optical image of an *α*‐MnSe flake, with a corresponding horizontal dimension. d) A characteristic AFM image with a cross‐sectional analysis. e) XRD patterns of the obtained *α*‐MnSe on mica substrate (black line), and the standard XRD patterns of *α*‐MnSe (blue line) and mica substrate (red line). XPS spectra of f) Mn 2P and g) Se 3d.

*α*‐MnSe possesses a rock‐salt‐type structure, and belongs to cubic *Fm*‐3_*m* (225) space group with the unit cell parameters *a* = *b* = *c* = 5.224 Å.^[^ ^17^, ^19^ ^]^ The crystal structure of *α*‐MnSe is visualized by the illustration on the right in **Figure** **1**a, of which Mn atoms and Se atoms are alternately connected. According to this arrangement, the above two kinds of atoms are connected by chemical bonds to extend in space, and no layered structure is present in any crystal direction, which indicates its nonlayered structural property. 2D *α*‐MnSe flakes were synthesized by space‐confined CVD at ambient pressure, which was a benefit in the formation of ultrathin 2D materials. MnCl_2_ powder and Se powder were adopted as the precursor of Mn source and Se source, respectively, and a mixed gas containing argon (Ar) and hydrogen (H_2_) was introduced as carrier gas. Stacked mica substrates were used to collect samples, which provide a limited space to effectively reduce the concentration of evaporation source, and further adjust the reaction mechanism, benefiting the formation of ultrathin 2D materials with high crystalline quality, as confirmed in Figure S1, Supporting Information.^[^ ^20^ ^]^ Moreover, the morphology, domain size, thickness, and nucleation density of *α*‐MnSe crystals can be adjusted by controlling synthesis parameters (growth temperature, growth time, and the flow of H_2_), as described in Figure S2, Supporting Information, and the optimal preparation parameters have been obtained, as stated in the later Experimental Section.

The optical microscopy images of obtained *α*‐MnSe flakes are displayed in the Figure 1b,c, of which the domain sizes of most single crystals are over 20 µm, and the largest domain size is ≈62 µm. The corresponding atomic force microscope (AFM) image of *α*‐MnSe flake is shown in Figure 1d, and the attached height profiles confirmed sample thickness to be ≈5.9 nm. Furthermore, through precisely adjusting synthesis parameters, the thinnest sample obtained so far is 0.9 nm (Figure S3, Supporting Information). Other AFM images of *α*‐MnSe flakes with different thicknesses and a typical scanning electron microscope (SEM) image are also displayed in Figures S4 and S5, Supporting Information. According to previous reports, Mn atoms and Se atoms can be combined into substances with multiple element ratios and multiple phases.^[^ ^21^ ^]^ To identify the crystal structure of obtained sample, X‐ray diffraction (XRD) patterns were illustrated in Figure 1e, and a series of peaks were revealed, of which (111) peak at 28.29° and (222) peak at 58.47° are indexed to the standard *α*‐MnSe patterns (PDF NO. 11‐0683), and the other peaks are attributed to mica substrates. The absence of signals of original composition or impurity phases verifies the acquisition of pure *α*‐MnSe crystals and single‐crystal nature, and the preferential growth orientation (111) plane is also confirmed. Based on this, the top view and side view of *α*‐MnSe (111) crystal plane are presented in Figure S6, Supporting Information, which described hexagonal arrangement of the atoms and a single unit cell thickness between the top and bottom layer (9.55 Å), respectively, confirming the thickness of the above‐mentioned thinnest sample is approximately equal to one unit cell thickness. X‐ray photoelectron spectroscopy (XPS) spectra were employed to further investigate surface elemental composition and chemical states of the synthesized samples. As shown in Figure 1f,g, the peaks at 653.46 and 641.48 eV binding energies can be attributed to Mn 2p_1/2_ and 2p_3/2_, and the other two peaks at 645.20 and 658.02 eV may suggest the characteristics of Mn^2+^ and surface weak oxidation, respectively.^[^ ^22^ ^]^ Whereas, the peaks at 55.16 and 53.92 eV binding energies can be assigned to Se 3d_5/2_ and 3d_3/2_, and the peaks at 55.98 and 53.19 eV may indicate the weak oxidation of surface layer, respectively.^[^ ^18b^ ^]^

**Figure 2:**
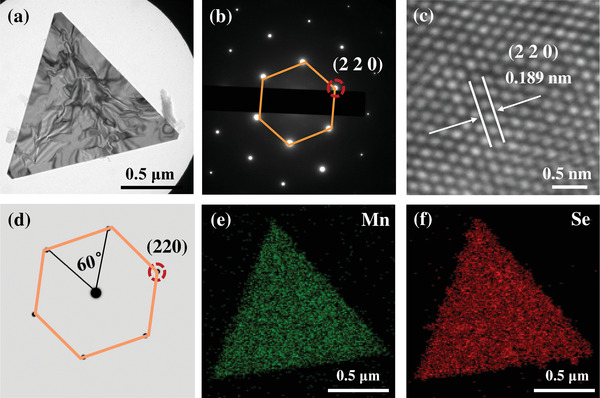
TEM characterizations of as‐synthesized *α*‐MnSe flake. a) Low‐magnification TEM image, b) SAED patterns, and c) the corresponding HRTEM image of *α*‐MnSe flake in (a). d) FFT patterns simulated by Crystal Maker, in accordance with the observed diffraction patterns in (b). e) Mn and f) Se elemental imaging of *α*‐MnSe flake.

To further reveal the detailed crystal structure and elemental composition of as‐grown *α*‐MnSe flakes, transmission electron microscopy (TEM) tests were carried out on the sample transferred on a copper grid via the polymers‐mediated dry transfer method.^[^ ^23^ ^]^ The low‐magnification TEM micrograph of *α*‐MnSe nanosheet was shown in **Figure** **2**a, which demonstrated its regular triangle morphology, and the apparent wrinkle on the flake may be derived from residual glue from the dry‐transfer process mentioned above. Then, selected area electron diffraction (SAED) patterns (Figure 2b) were obtained to determine the phase structure, of which the clear sixfold symmetry of diffraction patterns indicated its single‐crystal nature, and the equal interplanar distance verified that crystal plane family is cubic phase. Subsequently, a high‐resolution TEM image was obtained (Figure 2c), from which lattice fringes were clearly resolved and hexagonal arrangement of the atomic columns were present, confirming high crystallinity of the obtained crystals, and the observed average interplanar spacings is 0.189 nm, consistent with the distance of (220) interplanar planes. Based on CrystalMaker software, the calculated fast Fourier transforms diffraction patterns along [−111] direction was obtained, which is in favor of SAED data, as shown in Figure 2d. Besides, the uniform distribution of Mn and Se element in the crystal were also confirmed, as shown in the elemental maps of energy‐dispersive X‐ray (EDX) spectroscopy (Figure 2e,f). Moreover, the atomic ratio of Mn and Se elements was confirmed in the EDX spectrum, approximately equal to 1:1 (Figure S7, Supporting Information), demonstrating the obtained sample to be MnSe again. Therefore, we believe that the synthesized samples are cubic *α*‐MnSe with relatively high crystalline quality.

**Figure 3:**
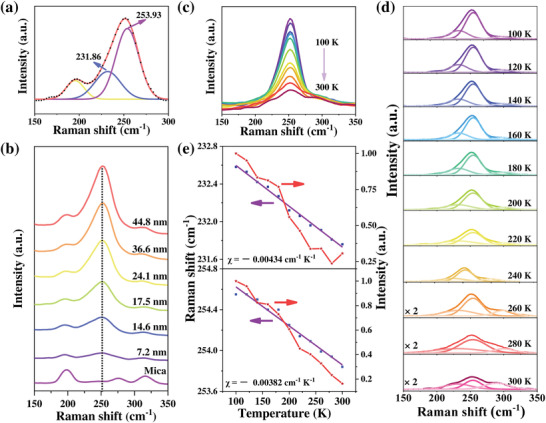
Raman characterizations of 2D *α*‐MnSe flakes. a) A typical Raman spectrum of 17.5‐nm‐thick *α*‐MnSe flake on mica substrate, and the corresponding peak splitting results. b) Thickness‐dependent Raman spectra of *α*‐MnSe flakes on mica. c) Temperature‐dependent Raman spectra of 17.5 nm‐thick *α*‐MnSe flakes transferred on SiO_2_ substrate, and d) the corresponding peak splitting results. e) The peak intensity and peak position varying with temperature from 100 to 300 K.

Raman spectra were usually recorded to understand the information on lattice vibration mode and phase structure.^[^ ^24^ ^]^ Therefore, a detailed investigation on the Raman spectrum of 2D *α*‐MnSe on mica substrate was presented. First, a typical Raman spectrum of 17.5‐nm‐thick *α*‐MnSe flake on mica excited with 532 nm laser was present in **Figure** **3**a. Excluding the peaks of mica substrate at 195 cm^−1^, a single peak located at 251.74 cm^−1^ was observed. Due to the asymmetry of peak shape, the prominent peak was further split into two peaks located at 231.86 and 253.93 cm^−1^, which were assigned to "longitudinal optical" (LO) mode and "transverse optical + longitudinal acoustic" (TO + LA) mode, respectively, arising from lattice vibrations.^[^ ^16^ ^]^ Here, the summation bands may imply crystal imperfections and the existence of defect structures inside the crystal.^[^ ^15^, ^16^ ^]^ Raman spectra of as‐grown *α*‐MnSe flakes on mica substrate depending on the sample thickness were obtained in Figure 3b, and the corresponding deconvoluted Raman spectra are shown in Figure S8, Supporting Information. The abovementioned two peaks can be observed in all selected samples with thickness varying from 7.2 to 44.8 nm. The peak intensities exhibit obvious thickness‐dependent characteristics, and they become stronger with the increasing sample thickness. On the contrary, the peak positions show no significant deviation, which may be related to the firm chemical bonds inside 2D *α*‐MnSe flakes, revealing non‐van der Waals structure of *α*‐MnSe.^[^ ^18b^ ^]^

To deeply understand thermal conductivity, thermal deformation, and thermal expansion behavior of 2D *α*‐MnSe flakes, temperature‐dependent Raman spectra of 17.5 nm‐thick *α*‐MnSe flake on SiO_2_ substrate were collected in Figure 3c, and the corresponding peak splitting results, and the peak intensity and peak position varying with temperature were extracted in Figure 3d,e. Raman spectra were recorded by gradual cooling from 300 to 100 K. It can be concluded that both the “LO” mode and “TO + LA” mode of 2D *α*‐MnSe flakes displayed a redshift with the gradual temperature rise. The peak positions varying with temperature can be expressed as a first‐order temperature coefficient (*χ*) of the following equation:

(1) $$\omega T=\omega_{0}+\chi T$$

where *ω*_0_, *χ*, and *T* represent the peak position of the vibration mode at zero Kelvin, the temperature coefficient, and the tested Kelvin temperature, respectively. The fitted *χ* for “LO” mode and “TO + LA” mode are −0.00434 and −0.00382 cm^−1^ K^−1^, respectively, lower than most 2D layered materials, such as ReSe_2_ (179.4 cm^−1^, *χ* = −0.0118 cm^−1^ K^−1^) and GeSe_2_ (A_g_ mode, *χ* = −0.0095 cm^−1^ K^−1^).^[^ ^25^ ^]^ Here, the lower fitting coefficients indicate that thermal changes have little effect on phonon frequencies, derived from the strong chemical bonds inside this nonlayered material.

In addition, with the decreasing temperature, the peak intensities of the two modes mentioned above show a gradual increase, which may be attributed to the resonance enhancement effect, probably related to the *T*_*N*_ obtained by the cooling process less than 100 K.^[^ ^16^ ^]^ Below *T*_*N*_, the paramagnetic state will transform to the antiferromagnetic state, which can induce the energy relaxation of the Mn^2+^ (3d^5^) state of *α*‐MnSe, leading to the Mn^2+^ excitation band (A_1g_–T_2g_) transition from a paramagnetic state (2.335 eV) to an antiferromagnetic state (2.41 eV) at a higher energy level. In this work, Raman data were obtained by exciting the sample with a 532 nm (≈2.33 eV) laser, and laser energy may perfectly match with transition energy of A_1g_–T_2g_ at the antiferromagnetic state.^[^ ^18b^ ^]^ Therefore, when lowering the temperature from 300 to 100 K, resonance‐enhancing effects will occur in Raman spectra near the *T*_*N*_ (100 K), inducing the enhancement of Raman intensity.^[^ ^15b^ ^]^ In addition, dramatic change in the spectral intensity associated with magnetic phase transition was absent in the temperature‐dependent Raman spectroscopy, which may be related to the fact that the tested temperature range was not low enough to cover *T*_*N*_.

**Figure 4:**
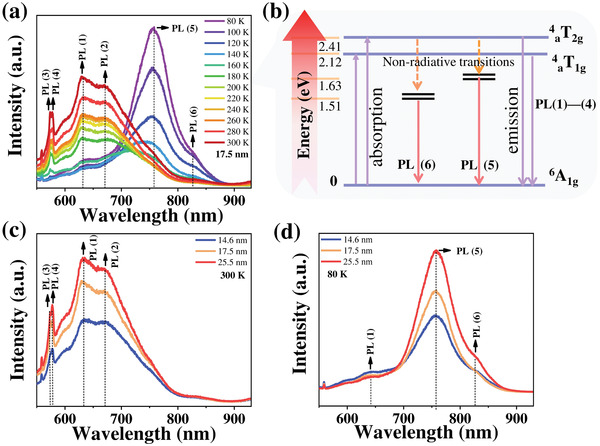
PL characterizations of 2D *α*‐MnSe flakes. a) Temperature‐dependent PL spectra collected from 80 to 300 K. b) Possible electron energy level diagram of *α*‐MnSe crystal. c) PL spectra of *α*‐MnSe flakes with various thicknesses at 300 K. d) PL spectra of *α*‐MnSe flakes with different thicknesses at 80 K.

Electronic structure and optical properties are sensitive to magnetic phase transition associated with spin ordering, magnons, and defect structures in 2D materials. Here, to explore the effect of spin ordering and magnons on emission properties and detect excitation states and defect states, temperature‐dependent PL spectra of as‐prepared 17.5‐nm‐thick *α*‐MnSe flake on SiO_2_ substrate were collected from 80 to 300 K in **Figure** **4**a. The PL spectra obtained at room temperature (300 K) displayed a broadband emission varying from 550 to 880 nm, close to the results of recorded polycrystalline *α*‐MnSe films prepared by a sintering technique.^[^ ^11b^ ^]^ As it can be seen, the primary emissions were located at 632.84 nm (1.96 eV, PL (1)) and 670.29 nm (1.85 eV, PL (2)), and the other two relatively weak bands were centered at 573.77 nm (2.16 eV, PL (3)) and 577.65 nm (2.14 eV, PL (4)), which are not assigned to bandgap emission (387.5 nm, ≈3.2 eV),^[^ ^11b^ ^]^ and may be attributed to the luminescence bands induced by direct internal ^4^_a_T_1g_→^6^A_1g_ transition, for the energy slightly lower and higher than well‐known internal d–d transitions of Mn^2+^ in diluted magnetic semiconductors, typically close to 2.12 eV.^[^ ^11^, ^26^ ^]^ Compared with the PL spectrum at 300 K, the primary emission bands have changed significantly at 80 K, and the peaks centered at 758.43 nm (1.63 eV, PL (5)) and 823.56 nm (1.51 eV, PL (6)) occupy the predominant position, which may be corresponding to radiated emissions from local energy levels, induced by perturbed Mn^2+^ states in interaction with magnons and defects.^[^ ^11b^ ^]^ According to the spectral intensity varying with temperature, it should be observed that the intensity of all the above six PL peaks show a significant change when passing across 160 K, which may be related to spin‐ordering‐induced magnetic phase transitions, and the temperature point (160 K) of drastic change is considered to be *T*_*N*_ for the heating process of 2D *α*‐MnSe, consistent with the reported data.^[^ ^18b^ ^]^ For magnons‐induced luminescence (PL (5)), the emission intensity decreases as the temperature rises within the low‐temperature range (<160 K) and disappears at higher temperatures (>160 K), which verifies its connection with spin ordering and only exists in the antiferromagnetic phase. In addition, the corresponding peak position shows a significant shift with increasing temperature below 160 K, which indicates that localized energy sublevels of Mn^2+^ transfer to higher energy levels, by the disturbance of magnons. For defects‐induced luminescence (PL (6)), the corresponding emission intensity also decreases as the temperature rises within the low temperature range (<160 K) and nearly equalizes with the intensity of the remaining emission peaks over 160 K, but the emission peak can be resolved up to 300 K, which indicated that the PL (6) emission band stayed visible in the paramagnetic phase. Peak intensity changing dramatically at 160 K suggested that it may be influenced by spin ordering, but not activated by magnons, similar to previous reports.^[^ ^11b^ ^]^ Peak intensity decreasing with the increasing temperature may be ascribed to the fact that as the temperature increases, the defect states will gradually be occupied by electrons, which leads to the consumption of more photogenerated carriers and dissipation of more phonons in the thermal radiation process. Then, the reduction in photon number leads to the decrease of radiation transfer process, and the PL intensity of emission induced by defects decreases.^[^ ^27^ ^]^ On the contrary, the strongest peaks at 300 K locked at 632.84 nm (1.96 eV, PL (1)) and 670.29 nm (1.85 eV, PL (2)) are hardly observed at low temperature (<160 K), and they strengthen gradually with the increasing temperature, and can be clearly observed over 180 K, which suggested that these emission bands are stronger in the paramagnetic state, and these emission intensities are likely to be greatly affected by spin ordering. As for the reason why it increases with temperature, it can be explained that as the temperature increases, the excitons trapped by localized states can be thermally activated into the delocalized states and recombine as free excitons or captured by the competing nonradiative decay channels, leading to the intensity increase of the free exciton emission.^[^ ^28^ ^]^

According to temperature‐dependent PL emission spectra, the possible electron energy level scheme was displayed in Figure 4b to explain the luminescence mechanism of *α*‐MnSe, and similar schemes have been observed in other Mn‐compounds with NaCl crystal structure.^[^ ^29^ ^]^ In *α*‐MnSe, when luminescence bands was excited, and energy was transferred by the “‘d–d”’ transition from the ^6^A_1g_ ground state into the ^4^_a_T_1g_ excited state of Mn^2+^ ions, in which “‘d–d”’ transitions are the mixture of Mn 3d^5^ and Se 4p^4^ states. Then, part of the excited Mn^2+^ energy was transferred to the ground state, forming luminescence bands (PL (1)‐PL (4)); another part of the excitation energy was transferred to localized energy levels in a nonradiative way, which derive from perturbed Mn^2+^ states in interaction with and magnons and defects. Further de‐excitation from these sublevels in a radiative way forms an emission band centered at 1.63 eV (PL (5)) and 1.51 eV (PL (6)), respectively. Moreover, the peak position and relative intensity of emission band from the sublevels usually vary depending on sample preparation (annealing time), crystallinity, and kinds of defects. In addition, the band at about 2.12 and 2.41 eV represents ^4^_a_T_1g_→^6^A_1g_ and ^4^_a_T_2g_→^6^A_1g_ transition, respectively, but the latter has not been observed in our experiment, because it is a weak emitting band and only exists in an ultralow temperature range (1.8–20 K).^[^ ^11b^ ^]^

Besides, typical PL spectra of as‐prepared *α*‐MnSe flakes with varying thickness at 300 and 80 K are presented in Figures 4c and 4d, respectively. The PL peak position does not change significantly with increasing thickness, derived from nonlayered structure; while, the emission intensity enhanced gradually with the increase of sample thickness both at 300 and 80 K, stemming from the increase of excited electrons number and the increase of magnons and defects concentration, respectively. It should be noted that although bandgap emission cannot be detected by our equipment, the strong emission intensity of the primary peak at low temperature implied that magnons‐ and defects‐induced emissions from localized energy levels are one of the main emission paths, which are of great significance to optoelectronic devices.^[^ ^30^ ^]^

Then, the electrical performance of *α*‐MnSe flake has been thoroughly investigated, based on construction of back‐gate FET on SiO_2_/Si substrate (Figure S9, Supporting Information). All the following electrical characterizations were carried out under room temperature and atmospheric conditions. The typical output characteristic curves and transfer characteristic curves are displayed in Figure S10a,b, Supporting Information, of which the symmetry changes of output characteristic curves imply that there is a near Ohmic contact at *α*‐MnSe‐electrodes interface. Moreover, the drain current (*I*_ds_) gradually decreases with the increasing gate voltage (*V*_g_), confirming *α*‐MnSe semiconductor with p‐type conductivity, and the corresponding on/off current ratio is ≈320, slightly inferior to some other 2D materials.^[^ ^31^ ^]^ Here, the p‐type conduction behavior was attributed to that the self‐contained manganese vacancies in *α*‐MnSe commonly play the role of acceptors, similar to 2D *α*‐MnS flakes.^[^ ^32^ ^]^ According to the formula *µ* = (Δ*I*_ds_/Δ*V*_g_) (*L*/(*W C*_i_ *V*_ds_)) (where channel length *L*, channel width *W*, and the gate capacitance *C*_i_ were 4, 4 µm, and 11.6 nF cm^−2^, respectively), the mobility of the device was deduced to be about 0.88 cm^2^ V^−1^ s^−1^ after being annealed in an inert atmosphere for enhancing electrode−2D material contacts (Figure S11, Supporting Information), close to the carrier mobility of 2D *γ*‑Ga_2_S_3_ and ReS_2_ flakes.^[^ ^20^, ^33^ ^]^ In addition, the carrier mobility and on/off ratio raised with increasing thickness, as shown in Figure S10c, Supporting Information. Here, the relatively general mobility may be ascribed to internal defects scattering and interfacial charge impurities scattering, further affecting the migration speed and conductivity of carriers. Inferior to the thicker samples, the thinner flake displayed lower carrier mobility and modulation ability, owning to the fact that within a certain thickness range, the *V*_g_ can effectively modulate the charge throughout the channel, but the relatively thin sample are more susceptible to the interfacial charged impurities scattering, consistent with previously informational results.^[^ ^34^ ^]^ Further increase of carrier mobility can be realized by improving film quality, optimizing contact and interface, including in situ using low temperature and vacuum measurement, channel encapsulation, and high k dielectrics, thus achieving reduction of scattering to carriers.^[^ ^35^ ^]^

**Figure 5:**
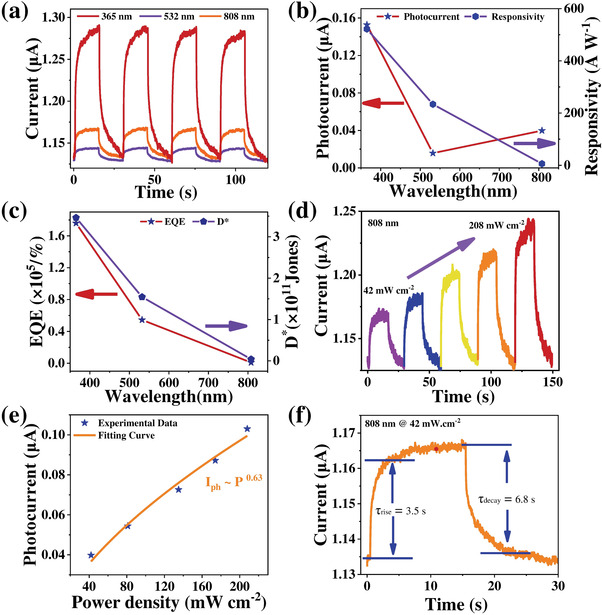
Optoelectronic properties of *α*‐MnSe‐based photodetector under room temperature and atmospheric conditions. a) Time‐resolved photoresponse of the device under dark and periodic illumination with various excitation wavelengths (365 nm @1.83 mW cm^−2^; 532 nm @0.42 mW cm^−2^; 808 nm @42 mW cm^−2^). b) *I*_ph_ and *R*_*λ*_, c) EQE and D* of the device at various excitation wavelengths. d) *I*−*T* curves of the device under 808 nm laser with various intensities at a bias voltage of 1 V. e) Photocurrent versus varying light intensity and the acquired fitting curve. f) Temporal response of the photodetector under 808 nm illumination.

Based on the above‐mentioned spin‐assisted or defects/magnons‐induced broadband optical properties and excellent electrical properties, 2D *α*‐MnSe flakes should exhibit excellent optoelectronic properties theoretically. Therefore, the photodetectors based on 2D *α*‐MnSe flake were constructed on mica substrates by a one‐step laser direct writing process, with Cr (10 nm)/Au (80 nm) metal as contact electrodes. Photoresponse properties were systematically studied under room temperature and atmospheric pressure, as summarized in **Figure** **5**. Considering the optical bandgap of *α*‐MnSe locked at 3.2 eV and detectable wide PL spectrum range (550–880 nm), typical lasers of 365, 532, and 808 nm were selected as the incident light sources. The corresponding current–time (*I*–*T*) cyclic curves and current–voltage (*I*–*V*) curves of 2D *α*‐MnSe photodetector under dark and illumination conditions are shown in Figure 5a and Figure S12, Supporting Information, from which an ultraviolet–near‐infrared broadband photoresponse was realized, similar to *α*‐MnSe film‐based device.^[^ ^36^ ^]^ It originates from the fact that the existence of defects and magnons varies the electron energy level of *α*‐MnSe and forms two groups of sublevels, and the electron transition between the defects/magnons and the ground state generates an ultrabandgap photoresponse under the illumination, in accordance with PL spectrum. In addition, the stability and reproducibility of 2D *α*‐MnSe‐based device under periodical illumination with different incident wavelengths were also demonstrated, confirming the reversibility of photoelectric conversion. Based on the same device sizes as the above FET devices, the corresponding photocurrent (*I*_ph_), *R*_*λ*_, external quantum efficiency (EQE), and D* under different incident light are calculated (Figure 5b,c), which represent the difference of the current under illumination and dark current, photon‐to‐electron conversion capability, the ability of a single photon to collect electrons, and the sensitivity of a photodetector with respect to dark current, respectively, as the main indexes for evaluating photodetector performance. According to the formula *I*_ph_ = *I*_on_ − *I*_dark_, *R*_*λ*_ = *I*_ph_/*PS* (where *I*_ph_, *I*_on_, *I*_dark_, *P*, and *S* are the photogenerated current, current under illumination, dark current, laser power density, and effective detection area, respectively); the device exhibits excellent photoresponse to 365 nm laser with a light intensity of 1.83 mW cm^−2^, with an *I*_ph_ up to 0.152 µA and a *R*_*λ*_ up to 521.8 A W^−1^ (Figure 5b). Here, the ultrahigh *R*_*λ*_ is superior to that of most 2D materials‐based devices, such as PbI_2_ and *α*‐MnS,^[^ ^37^ ^]^ which may be attributed to its inherent direct bandgap and the existence of defect energy level, leading to majority carriers circulating the circuits many times and minority carriers localized via by defects, and contributing to higher *R* *_λ_*. Based on the formula EQE = *hcR*_*λ*_/*eλ* and D* = *R*_*λ*_ *S* ^1/2^/(*2eI*_dark_)^1/2^ (where *h*, *c*, *R*_*λ*_, *e*, *λ*, *S*, and *I*_dark_ are Plank's constant, light velocity, responsivity, elementary charge, incident wavelength, effective detection area, and dark current, respectively), a maximum EQE of 1.76 × 10^5^% and D* of 3.46 × 10^11^ Jones for 365 nm illumination were further obtained (Figure 5c), superior to the device performance of other 2D materials.^[^ ^20^, ^38^ ^]^ In addition, the four key indicators mentioned above show a declining tendency when the incident wavelength undergoes a red shift, which confirms that photodetection performance exhibits an obvious wavelength‐dependent characteristic.

Based on the spin‐ordering‐related super‐bandgap broadband photoresponse of the device, we focus on the photodetection performance of the device under 808 nm illumination. To figure out the photoelectric conversion mechanism of *α*‐MnSe flakes, the dependence of photocurrent on 808 nm incident light intensity is displayed in Figure 5d, in which the enhanced laser power density leads to monotonically rising photocurrent. According to a power law (*I*_ph_ ∝ *P^*α*^*; *α* is a constant within the limits of 0–1, *P* is light power density), the curve of photocurrent depending on light intensity can be fitted with a factor of 0.63, as shown in Figure 5e, which confirmed the sublinear response behavior, attributed to the trap states induced by the defects in *α*‐MnSe flakes. Theoretically, few trap states will result in a linear dependence between photocurrent and optical power (*α* = 1), and the appearance of more non‐negligible trap states will result in a smaller fitting factor (*α* < 1). Here, the fitting factor 0.63 implies low concentration defects and trap states affect the generation and separation of photogenerated electron–hole pairs. At lower light power, the trap state will be filled with electrons separated from the photogenerated electron–hole pair. With a gradual increase of optical power, the number of electron–hole pairs increases significantly, and the trap states will be filled gradually; once the trap states reach saturation, excess electrons and holes will recombine quickly and not contribute to the photocurrent, consistent with other 2D materials.^[^ ^38a^ ^]^ The above‐mentioned sublinear behavior once again verified that the synthesized 2D *α*‐MnSe contains defects, in consistent with the data of the PL data. The temporal response of 2D *α*‐MnSe photodetector under 808 nm laser is shown in Figure 5f, which confirms the response time of 3.5 s and decay time of 6.8 s, extracted from 0% to 90% of the peak output value, respectively. Here, the response time is slower than that of 2D CdTe photodetector^[^ ^39^ ^]^ and 2D RhI_3_ photodetector.^[^ ^34^ ^]^ To further understand the internal essence of long carrier recombination time, lifetime information of *α*‐MnSe flake (Figure S13, Supporting Information) was obtained by adopting the time‐resolved and laser‐scanned confocal microscopy.^[^ ^40^ ^]^ Fitted by a specific exponential function, time‐resolved PL decay of carriers generates an intrinsic carrier lifetime of ≈0.98 ns, longer than previously reported data such as 2D MoS_2_ (few ps).^[^ ^41^ ^]^ This relatively long carrier life is probably related to crystal defects, and it can account for the slow recombination rate, contributing to the excellent performance of the photodetector. The detailed power‐density‐dependent performance indicators (*I*_ph_, *R*_*λ*_, EQE, and D*) under the irradiation of 808 nm laser are shown in Figure S14, Supporting Information. With the increase of optical power density, photocurrent was enhanced gradually, and the remaining three performance indexes show the opposite trend. In addition, optoelectronic properties of *α*‐MnSe device under the illumination of 365 nm laser were also summarized and discussed in Figure S15, Supporting Information, similar to the performance of device excited by 808 nm laser.

**Figure 6:**
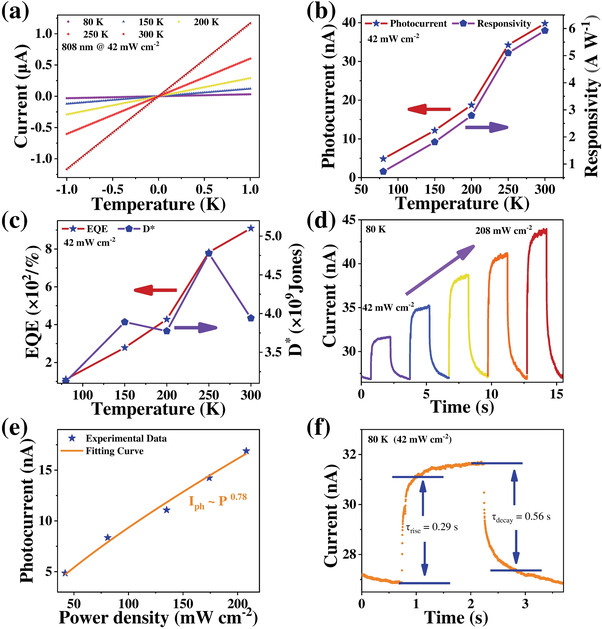
Temperature‐dependent optoelectronic properties of *α*‐MnSe device under the illumination of 808 nm laser. a) Temperature‐dependent current of the device under 808 nm laser. b) *I*_ph_ and *R*_*λ*_, c) EQE and D* of an *α*‐MnSe photodetector under 808 nm incident light in 80–300 K. d) Light‐intensity‐dependent photoresponse at a bias voltage of 1 V at 80 K. e) Photocurrent versus light intensity and the obtained fitting curve at 80 K. f) Temporal response of *α*‐MnSe‐based photodetector at 80 K.

In order to further determine whether antiferromagnetic spin ordering leaves footprints on photodetection performance, temperature‐dependent optoelectronic performance under 808 nm laser irradiation and ultravacuum conditions were recorded in the heating process and summarized in **Figure** **6**. Temperature‐dependent current under 808 nm laser and dark conditions are shown in Figure 6a and Figure S16, Supporting Information. Both the current under illumination and dark conditions increased significantly with the rising temperature, which were ascribed to suppression of thermal emission carriers at low temperature, similar to 2D *α*‐MnS.^[^ ^37a^ ^]^ Then, *I*_ph_ and *R*_*λ*_, EQE, and D* varying with temperature were extracted and depicted in Figure 6b,c. With the temperature rising from 80 to 300 K, *I*_ph_ show a gradual increase, confirming that the current under illumination is more sensitive to temperature than dark current. Similarly, *R*_*λ*_, EQE, and D* also show an obvious decrease trend with decreasing temperature. Here, dramatic change of photodetection performance was not obvious, which may be derived from that the temperature's influence on the resistance masks the influence of antiferromagnetic spin ordering. Subsequently, the performance of the device at 80 K was investigated in detail. Compared with the results at 300 K, in addition to the reduced photocurrent and dark current, the photodetector also exhibits an improved photocurrent–power density fitting factor (0.78), and an accelerated response speed (*τ*_rising_ = 0.29 s and *τ*_decay_ = 0.56 s), as present in Figure 6d–f. These improvements may be attributed to the change of spin ordering and the suppression of defects induced by low vacancy density and fewer trap states at low temperature,^[^ ^42^ ^]^ confirming that spin ordering and the trap states caused by the defects have an important influence on photodetection performance, different from other previous reports.^[^ ^43^ ^]^ In addition, all the performance of our 2D *α*‐MnSe‐based photodetector were extracted to be compared with those of other previously reported 2D‐based devices, as shown in Table S1, Supporting Information. The ultrathin *α*‐MnSe‐based photodetector in our work exhibits ultraviolet–near‐infrared broadband detection, ultrahigh responsivity, and detectivity, superior to most reported 2D‐devices, which verified that 2D *α*‐MnSe has a promising application prospect in optoelectronic field.

## 3 Conclusion

In summary, the controlled preparation of ultrathin 2D *α*‐MnSe flakes via a space‐confined CVD method has been demonstrated, and the influence of spin ordering and magnons on their optical/optoelectronic properties was systematically explored in this work. The prepared 2D *α*‐MnSe nanosheets exhibit a lateral size up to 62 µm and a thickness as thin as 0.9 nm. The spin ordering induces the emergence of a sharp change point in the PL spectra but absence in the Raman spectrum, due to the existence of thermal hysteresis. The existence of magnons and defects in 2D *α*‐MnSe crystal was confirmed by low‐temperature PL spectra, which endow itself with a broadband luminescence from 550 to 880 nm, an ultraviolet–near‐infrared broadband photoresponse, and excellent ultraviolet detection performance (an excellent *R*_*λ*_ of 521.8 A W^−1^, an ultrahigh EQE of 1.76 × 10^5^%, and an outstanding D* of 3.46 × 10^11^ Jones, @365 nm laser), outperforming most 2D materials. In addition, the device exhibits a superior photocurrent‐optical power fitting factor and response time at 80 K to those at 300 K, which may be attributed to the change of spin ordering and the existence of trap states associated with defects. All the above results indicate that this novel 2D magnetic semiconductor *α*‐MnSe is a suitable carrier for studying the magneto‐optical and magneto‐optoelectronic properties, and possesses tremendous potential in high‐performance broadband photodetector.

## 4 Experimental Section

### Synthesis of 2D *α*‐MnSe Flakes

Ultrathin 2D *α*‐MnSe flakes were synthesized via a space‐confined CVD method. As shown in schematic representation in Figure 1a, MnCl_2_ (99.999%, Alfa) source was placed in the center zone of a tube furnace, and Se (99.9%, Alfa) powder were located at upstream zone, and mica substrates were placed downstream to collect *α*‐MnSe flakes. The preparation procedure was as follows: first, high purity Ar was pushed through the furnace; then the quartz tube was heated to 660 °C and held for 10 min, and 120 sccm Ar and 5 sccm H_2_ flow were pumped into the quartz tube at the same time; subsequently, the furnace was cooled to room temperature after reaction.

### Characterization and Measurement

The surface morphologies and thicknesses were studied by an AFM (Dimension Fast Scan, Bruker), the microstructure and the composition were confirmed through a TEM (Tecnai G2 F30, FEI), EDX, XRD (D8QUEST, Bruker), and XPS (ESCALab250 Xi, Thermo Fisher Scientific), and Raman and PL spectra were carried out on a confocal Raman/PL system (Alpha 300R, WITec).

### Device Fabrication and Characterization

The laser direct writing (MicroWriter ML Baby‐plus, Durham) and electron beam lithography (FEI Quanta 650 SEM and Raith Elphy Plus) were used to construct the device of two‐probe electrodes (photodetector) and three‐probe electrodes (FET device), separately; and Cr (10 nm) and Au (80 nm) were thermally deposited on the sample as electrodes (Nexdep, Angstrom Engineering). Annealing the device at 200 °C under high purity Ar gas was applied to enhance the electrode–sample contact. A semiconductor analyzer (B1500A, Agilent) with a 365, 532, and 808 nm laser and a probe station (CRX‐6.5K, Lake Shore) were used to measure the electric and photoelectric performances.

## Conflict of Interest

The authors declare no conflict of interest.

## Supporting information

Supporting Information

## Data Availability

The data that support the findings of this study are available from the corresponding author upon reasonable request.
