# Supplementary material for: Spin Ordering Induced Broadband Photodetection Based on Two‐Dimensional Magnetic Semiconductor α‐MnSe
Source: Adv Sci (Weinh). 2022 Jun 5;9(22):2202177. doi: 10.1002/advs.202202177 (PMC9353471; doi:10.1002/advs.202202177)
Supplement: Supplementary file 1 — Supporting Information [file ADVS-9-2202177-s001.pdf]

((Supporting Information can be included here using this template))

Supporting Information

Spin Ordering Induced Broadband Photodetection Based on Two-Dimensional Magnetic Semiconductor α-MnSe

Nan Zhou, Zhimiao Zhang, Fakun Wang, Junhao Li, Xiang Xu, Haoran Li, Su Ding, Jinmei Liu, Xiaobo Li, Yong Xie, Rusen Yang, Ying Ma, and Tianyou Zhai**

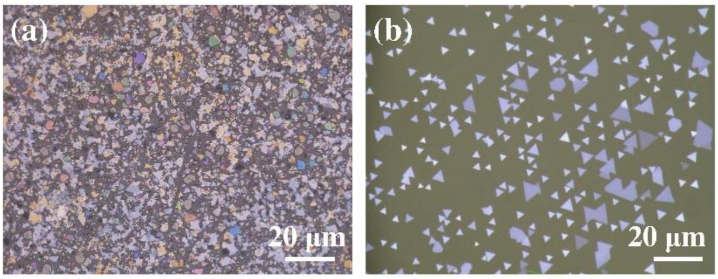

Figure S1. The comparison OM images of α-MnSe flakes obtained by (a) the non-confinement method (using a row of monolithic mica substrates to collect samples) and (b) the optimized space-confined CVD method, respectively.

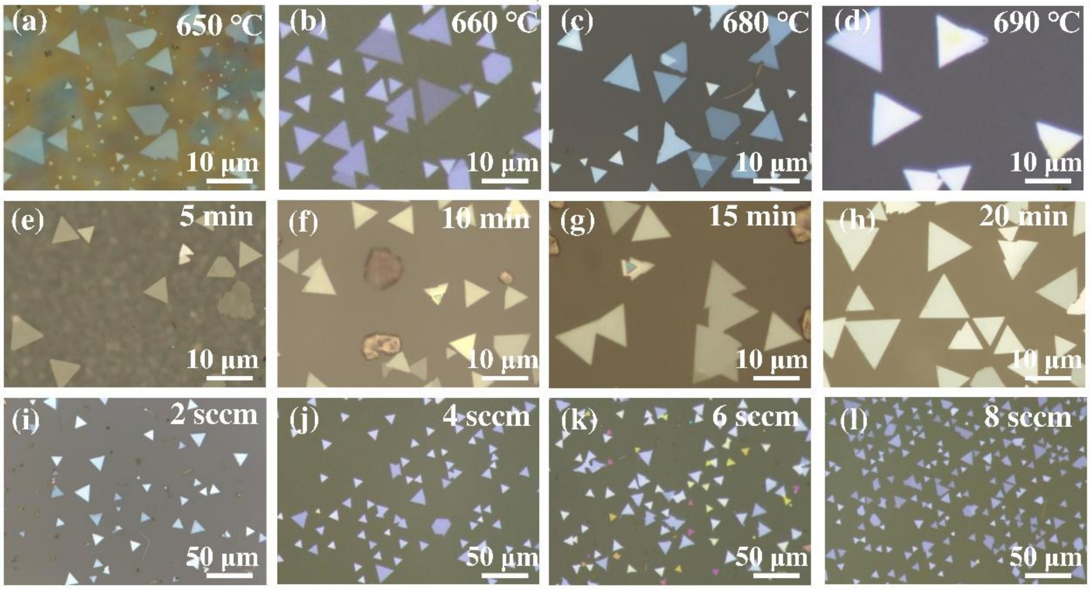

Figure S2. OM of the obtained α-MnSe flakes with different synthesis parameters, including the rising growth temperature (a-d), the extending growth time (e-h) and the increasing proportion of H_2 (on the basis of Ar flow being 50 sccm), respectively.

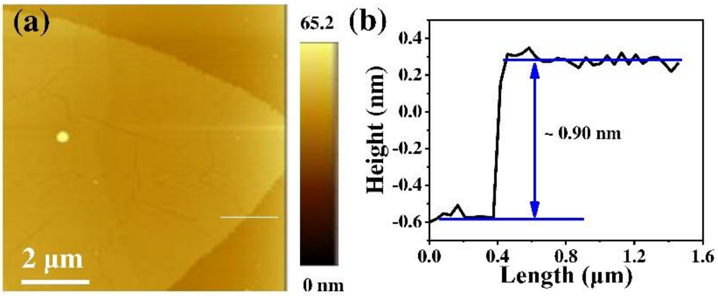

Figure S3. (a) The AFM image of the obtained thinnest α-MnSe flake on mica substrate, and (b) the corresponding height profile, verifying the synthesized sample can be as thin as 0.9 nm.

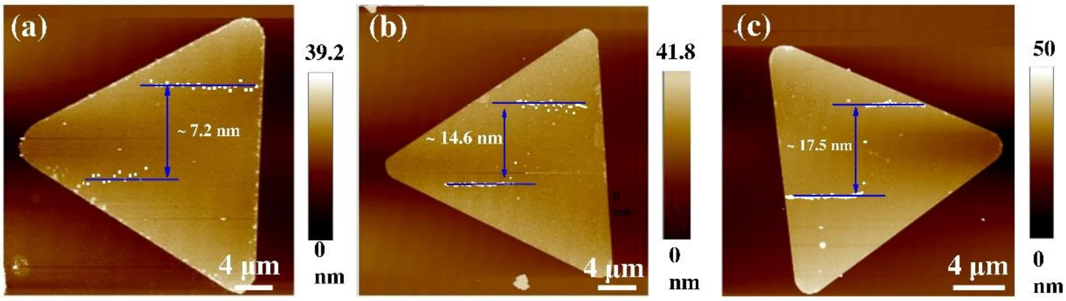

Figure S4. The typical AFM images of α-MnSe flakes with different thickness, and the associated height profiles.

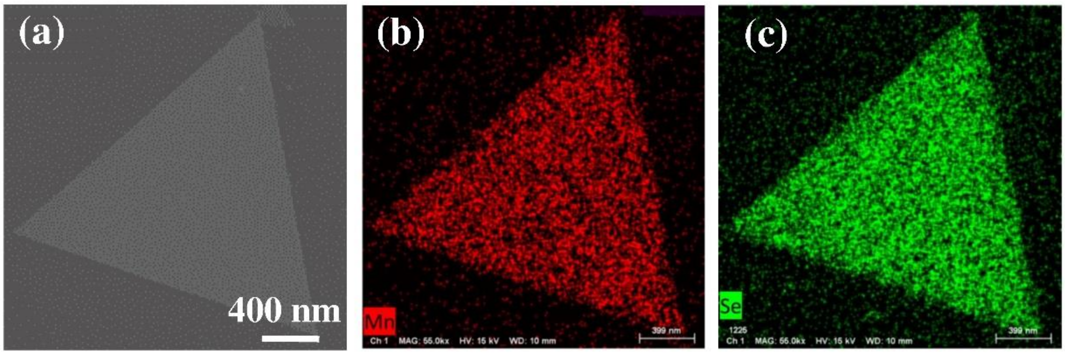

Figure S5. (a) A typical SEM image and (b, c) energy dispersive X-ray spectroscopy (SEM-EDX) of triangle-shaped α-MnSe flake.

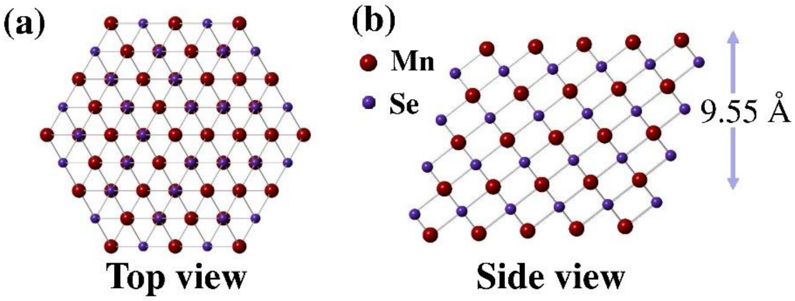

Figure S6. The top and side views of α-MnSe (111) crystal plane. Mn atoms are marked by red, and Se atoms are marked by blue.

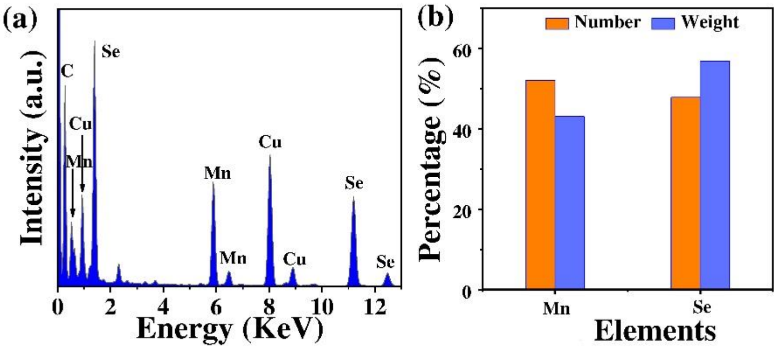

Figure S7. (a) The TEM-EDX analysis of α-MnSe triangular flakes on grids, and (b) the quantity ratio and weigh ratio of Mn and Se elements.

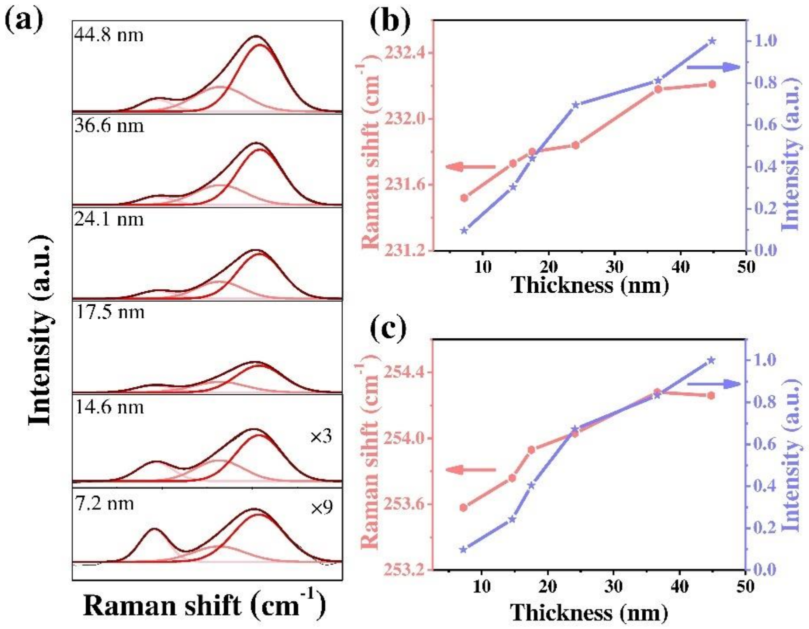

Figure S8. (a) The detailed Raman peaks splitting results of α-MnSe flakes with different thickness on mica in Figure 3b. (b, c) Thickness-dependent peak position and peak intensity of “LO” mode and “TO+LA” mode, respectively.

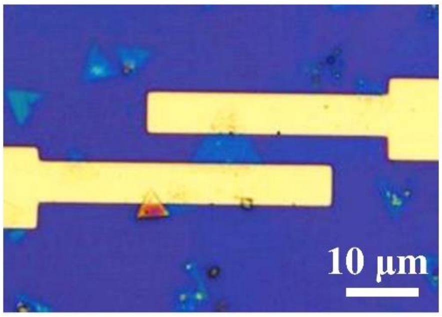

Figure S9. OM image of FET device based on 2D α-MnSe flake on SiO_2 substrate.

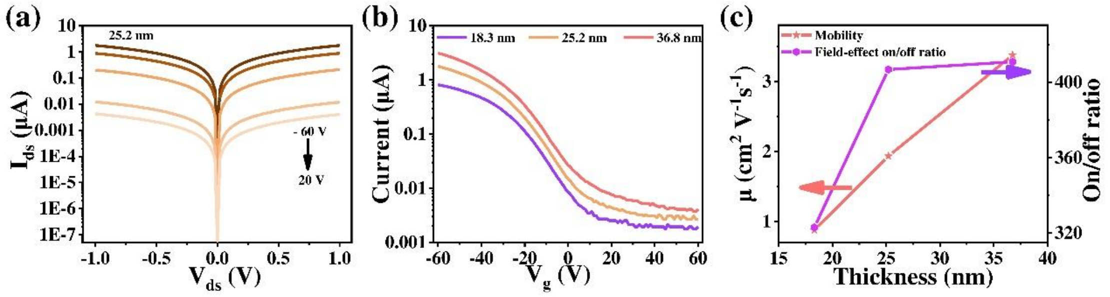

Figure S10. (a) Output characteristic curves (in logarithmic form) and (b) transfer characteristic curves (in logarithmic form) of FET device based on 2D α-MnSe flakes with different thickness. (c) the corresponding carrier mobility and field-effect on/off ratio.

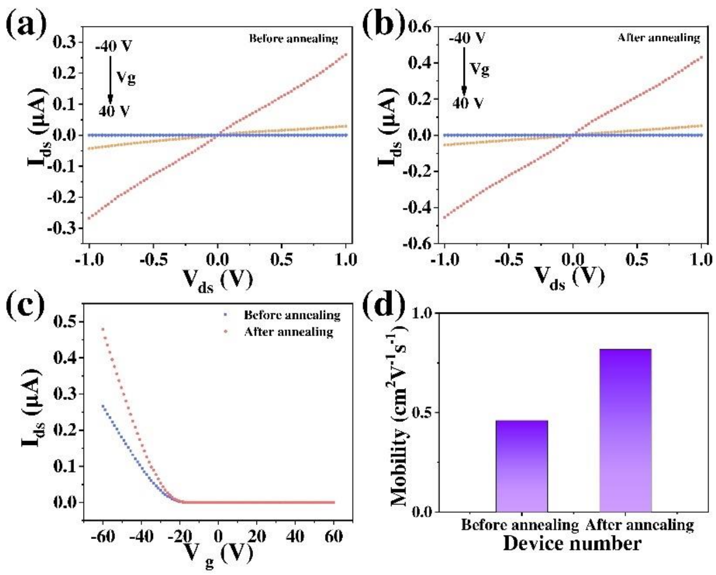

Figure S 11. (a-b) Output characteristic curve, (c) Transfer characteristic curve, and (d) carrier mobility of the device based on 2D α-MnSe flakes before and after annealing in an inert atmosphere.

All the FET devices we provided were annealed in an inert atmosphere before performance test, and the results in the **Figure S11** confirms that the improvement of device performance was limited through this method.

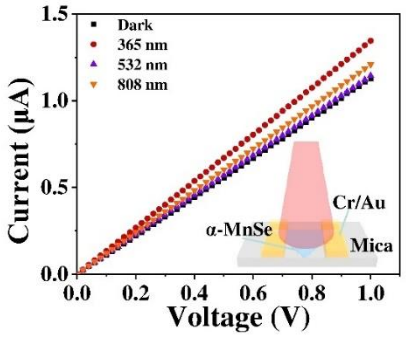

Figure S12. *I*−*V* characteristic curves of the device under dark and laser with various excitation wavelengths (365 nm @1.83 mW cm^{-2}; 532 nm @0.42 mW cm^{-2}; 808 nm @42 mW cm^{-2}).

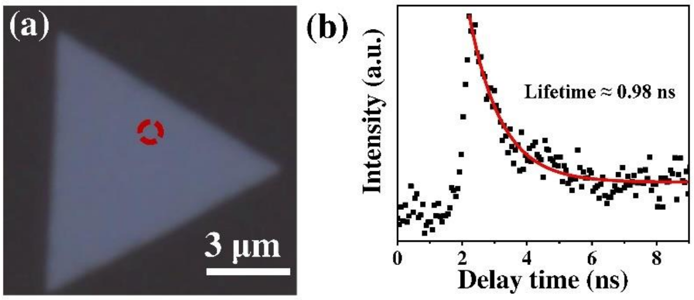

Figure S13. (a) OM image of α-MnSe flake. (b) A typical PL decay collected from the position marked by the red circle on α-MnSe flake in (a), showing a single exponential kinetics with a lifetime of 0.98 ns. The red solid line is the single exponential fit.

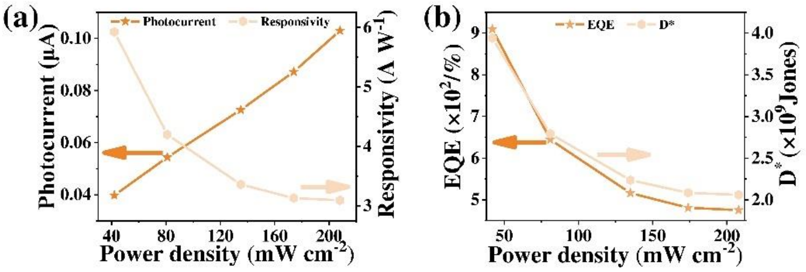

Figure S14. (a) I_{ph} and R_{λ}, (b) EQE and D^* of the device under 808 nm incident wavelengths with different light power density, respectively.

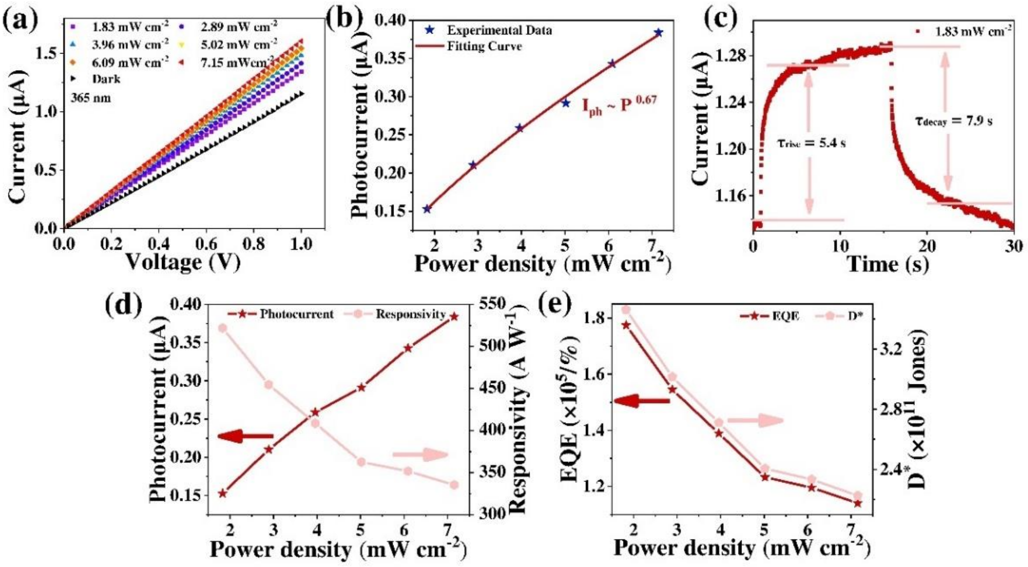

Figure S15. Optoelectronic properties of α-MnSe device under illumination of 365 nm laser. (a) I−V curves of the device under dark and 365 nm laser with various intensities. (b) Photocurrent versus varying light intensity and the acquired fitting curve. (c) Response and recovery curves of the photodetector. (d) I_{ph} and R_{λ}, (e) EQE and D^* of the device under 365 nm incident wavelengths with different light power density, respectively.

Optoelectronic properties of α-MnSe device under illumination of 365 nm laser were summarized in Figure S15. The relationship between photocurrent and light power were first surveyed, as exhibited in Figure S15a and S15b. The photocurrent was enhanced gradually as light intensity increased, and the corresponding fitting factor is 0.67, similar to that of the device excited by 808 nm laser. The obtained response time and recovery time are 5.4 s and 7.9 s, respectively. For photoconductors, high responsivity is often accompanied by slow response rate. Combined with the above-mentioned high responsivity (521.8 A W^{-1}), the relatively long response time may be attributed to trap states induced by the defects, in which

the photocurrent will be saturated only after the defect states are saturated, consuming a lot of time. The detailed power-density-dependent performance indicators (I_{ph}, R_λ, *EQE* and D^*) under the irradiation of 365 nm are also displayed, similar to the varying under 808 nm laser.

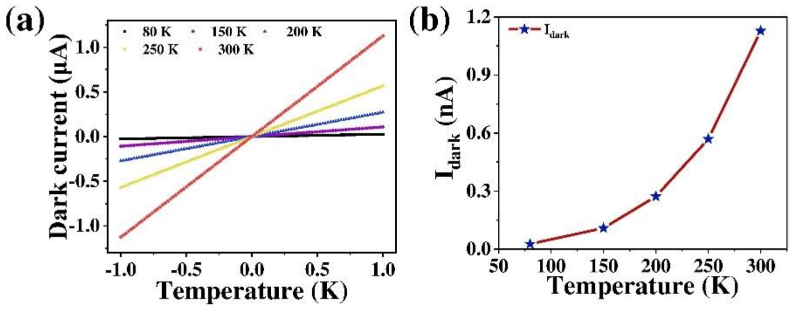

Figure S16. (a) Temperature-dependent dark current of an α-MnSe photodetector in 80-300 K, and (b) the extracted dark current value.

Table S1. Comparison of the photodetector performance with other reported 2D materials (CVD: chemical vapor deposition; ME: mechanical exfoliation.)

Photodetectors	Synthetic method	Detection Wavelength (nm)	R_λ ($A\ W^{-1}$)	EQE (%)	D* (Jones)	V_{bias}	Ref
$Ga_2In_4S_9$	CVD	330-800	111.9 (360 nm)	3.85×10^4 (360 nm)	2.25×10^{11} (360 nm)	5	1
α-MnS	CVD	473	139	\	3.2×10^{14}	2	2
CdTe	CVD	473	6×10^{-4}	\	10^9	1	3
Sb_2Se_3	CVD	300-950	4.32 (532 nm)	1001 (532 nm)	2.52×10^9 (532 nm)	2	4
Si_2Te_3	ME	405–1064	65 (405 nm)	14.01×10^3 (405 nm)	\	1	5
γ-Ga_2S_3	CVD	350-900	61.3 (350 nm)	2.17×10^4 (350 nm)	1.52×10^{10} (350 nm)	1	6
RhI_3	ME	980	11.5	\	2×10^{10}	1	7
α-MnSe	CVD	450	4.73×10^{-3}	\	1.31×10^{10}	2	8
α-MnSe	CVD	365, 532, 808	521.8 (365 nm)	1.76×10^5 (365 nm)	3.46×10^{11} (365 nm)	1	This work

REFERENCES

[1] F. K. Wang, T. Gao, Q. Zhang, Z.-Y. Hu, B. Jin, L. Li, X. Zhou, H. Q. Li, G. V. Tendeloo, T. Y. Zhai, *Adv. Mater.* **2019,** *31*, 1806306.

[2] N. N. Li, Y. Zhang, R. Q. Cheng, J. J. Wang, J. Li, Z. X. Wang, M. G. Sendeku, W. H. Huang, Y. Y. Yao, Y. Wen, J. He, *ACS Nano* **2019**, *13*, 12662.

[3] N. N. Li, R. Q. Cheng, Y. Wen, L. Yin, F. M. Wang, F. Wang, K. L. Liu, T. A. Shifa, J. Li, C. Jiang, Z. X. Wang, J. He, *Adv. Mater.* **2017,** *29*, 1703122.

[4] M. Zhao, J. W. Su, Y. Zhao, P. Luo, F. K. Wang, W. Han, Y. Li, X. T. Zu, L. Qiao, T. Y. Zhai, *Adv. Funct. Mater.* **2020**, *30*, 1909849.

[5] J. W. Chen, C. Y. Tan, G. Li, L. J. Chen, H. L. Zhang, S. Q. Yin, M. Li, L. Li, G. H. Li, *Small* **2021**, *17*, 2006496.

[6] N. Zhou, L. Gan, R. S. Yang, F. K. Wang, L. Li, Y. C. Chen, D. H. Li, T. Y. Zhai, *ACS Nano* **2019,** *13*, 6297.

[7] F. K. Wang, Z. Zhang, Y. Zhang, A. Nie, W. Zhao, D. Wang, F. Q. Huang, T. Y. Zhai, *Adv. Mater.* **2020,** *32*, 2001979.

[8] Z. C. Zhang, B. Zhao, D. Y. Shen, Q. Y. Tao, B. Li, R. X. Wu, B. L. Li, X. D. Yang, J. Li, R. Song, H. M. Zhang, Z. W. Huang, Z. W. Zhang, J. Y. Zhou, Y. Liu, X. D. Duan, *Small Struct.* **2021,** *2*, 2100028.
